# Assessing and mapping language, attention and executive multidimensional deficits in stroke aphasia

**DOI:** 10.1093/brain/awz258

**Published:** 2019-09-05

**Authors:** Rahel Schumacher, Ajay D Halai, Matthew A Lambon Ralph

**Affiliations:** 1 MRC Cognition and Brain Sciences Unit, University of Cambridge, Cambridge, UK; 2 Department of Neurology, Inselspital, Bern University Hospital, and University of Bern, Bern, Switzerland; 3 Neuroscience and Aphasia Research Unit, School of Biological Sciences, University of Manchester, Manchester, UK

**Keywords:** aphasia, executive functions, attention, principal components, univariate and multivariate brain-behaviour mapping

## Abstract

There is growing awareness that aphasia following a stroke can include deficits in other cognitive functions and that these are predictive of certain aspects of language function, recovery and rehabilitation. However, data on attentional and executive (dys)functions in individuals with stroke aphasia are still scarce and the relationship to underlying lesions is rarely explored. Accordingly in this investigation, an extensive selection of standardized non-verbal neuropsychological tests was administered to 38 individuals with chronic post-stroke aphasia, in addition to detailed language testing and MRI. To establish the core components underlying the variable patients’ performance, behavioural data were explored with rotated principal component analyses, first separately for the non-verbal and language tests, then in a combined analysis including all tests. Three orthogonal components for the non-verbal tests were extracted, which were interpreted as shift-update, inhibit-generate and speed. Three components were also extracted for the language tests, representing phonology, semantics and speech quanta. Individual continuous scores on each component were then included in a voxel-based correlational methodology analysis, yielding significant clusters for all components. The shift-update component was associated with a posterior left temporo-occipital and bilateral medial parietal cluster, the inhibit-generate component was mainly associated with left frontal and bilateral medial frontal regions, and the speed component with several small right-sided fronto-parieto-occipital clusters. Two complementary multivariate brain-behaviour mapping methods were also used, which showed converging results. Together the results suggest that a range of brain regions are involved in attention and executive functioning, and that these non-language domains play a role in the abilities of patients with chronic aphasia. In conclusion, our findings confirm and extend our understanding of the multidimensionality of stroke aphasia, emphasize the importance of assessing non-verbal cognition in this patient group and provide directions for future research and clinical practice. We also briefly compare and discuss univariate and multivariate methods for brain-behaviour mapping.

## Teaser

Despite being potentially crucial for performance, recovery and rehabilitation, non-verbal cognitive functions have not been investigated comprehensively in patients with post-stroke aphasia. By administering a battery of tests of attention and executive function, Schumacher *et al.* identify six orthogonal non-verbal and language components that are associated with separable structural correlates.

## Introduction

There is a growing understanding that a left hemispheric stroke leading to impairments in language processing—aphasia—often also affects other cognitive functions, such as attention or executive functions (Glosser and Goodglass, 1990; Helm-Estabrooks, 2002; Jefferies and Lambon Ralph, 2006; Murray, 2012; Villard and Kiran, 2017) and it has been shown that impairments in these cognitive functions play an important role in aphasia recovery and rehabilitation (Fillingham *et al.*, 2005; van de Sandt-Koenderman *et al.*, 2008; Lambon Ralph *et al.*, 2010; Brownsett *et al.*, 2014; El Hachioui *et al.*, 2014; Geranmayeh *et al.*, 2017; Simic *et al.*, 2019). The occurrence and patterns of non-verbal cognitive dysfunctions in patients with aphasia, the relationship between non-verbal and language impairments, and their structural correlates have been examined separately in some studies. To date, however, no investigation has undertaken a detailed behavioural assessment of both verbal and non-verbal performance or combined this with structural imaging data.

A handful of previous behavioural studies have examined non-verbal cognition in patients with aphasia, but did so either with a narrow focus, for instance investigating the impact of domain-general executive dysfunctions on semantic cognition (Thompson *et al.*, 2018), or on a rather general level with findings based on composite scores (Helm-Estabrooks, 2002), a few standardized tests per domain (Kauhanen *et al.*, 2000; Fucetola *et al.*, 2009; El Hachioui *et al.*, 2014; Lee and Pyun, 2014; Marinelli *et al.*, 2017; Wall *et al.*, 2017) or experimental tasks (Villard and Kiran, 2015; Kuzmina and Weekes, 2017). This limited test selection stands in contrast to research efforts with healthy participants or other patient populations that have explored the nature of multiple components within attention and executive function (Mirsky *et al.*, 1991; Miyake *et al.*, 2000; Friedman and Miyake, 2017). One study including patients with aphasia used a broad range of attention assessments and indeed found that aspects of attention differed with respect to their predictive power regarding language function (Murray, 2012).

Another limitation of existing studies is that patient performance is often reported on a group level only (Glosser and Goodglass, 1990; Kauhanen *et al.*, 2000; El Hachioui *et al.*, 2014; Lee and Pyun, 2014; Naranjo *et al.*, 2018) and information about the prevalence of impaired performance based on normative data is seldom available or incomplete. This information is, however, of clinical significance and relevant when performance in different aspects of cognitive functioning is to be compared.

Underlying patterns in impaired and preserved abilities of heterogeneous patient populations can be extracted using data reduction techniques, such as principal component analysis (PCA) (Kummerer *et al.*, 2013; Butler *et al.*, 2014; Mirman *et al.*, 2015; Halai *et al.*, 2017; Lacey *et al.*, 2017). Applied to large, detailed datasets containing language measures and a handful of executive function assessments, a previous study of chronic post-stroke aphasia found three principal components (phonology, semantics, executive function) underlying participants’ performance (Butler *et al.*, 2014), which was supplemented by a fourth speech quanta component (the quantity of speech produced in connected-speech tasks) in a subsequent study (Halai *et al.*, 2017). One major advantage of data-driven approaches is that they can accommodate for the fact that multiple processes underlie performance in any given test (e.g. naming requires preserved visual perception, semantics, phonology and motor articulation) and no test is a pure measure of single cognitive/language processes. Indeed, sensibility regarding the linguistic demands of any test is particularly high within the field of aphasia. These concerns are usually expressed in the sense that impaired language functions may interfere with testing of other cognitive domains (Keil and Kaszniak, 2002), and more rarely the other way around (Heuer *et al.*, 2017). Data-driven approaches offer a formal method to establish the mutual influences of language and non-verbal ability on test performance.

Based on studies with healthy controls and various neurological populations, a bilateral fronto-cingulo-parietal network is known to be involved in attention and executive function processes (Miller and Cohen, 2001; Duncan, 2010; Niendam *et al.*, 2012; Petersen and Posner, 2012; Fedorenko *et al.*, 2013; Power and Petersen, 2013) but little is known about the structural correlates of attentional and executive dysfunctions in patients with aphasia. Recent research combining data-driven decomposition of behavioural assessment with neuroimaging data, has revealed the structural correlates of behavioural performance in patients with aphasia (Kummerer *et al.*, 2013; Butler *et al.*, 2014; Mirman *et al.*, 2015; Halai *et al.*, 2017; Lacey *et al.*, 2017). While extracting clear brain-behaviour relationships for various aspects of language, these studies struggled to find significant associations of tissue integrity with scores on executive function (but see Lacey *et al.*, 2017), either because non-language assessment was not included (Kummerer *et al.*, 2013; Mirman *et al.*, 2015) or assessment coverage was too limited (Butler *et al.*, 2014; Halai *et al.*, 2017).

In addition to the form and analysis of patients’ behavioural assessment, the approach to mapping brain-behaviour relationships could also be critical. Univariate approaches, such as voxel-based lesion-symptom mapping (VLSM) (Bates *et al.*, 2003) and voxel based correlational methodology (VBCM) (Tyler *et al.*, 2005), are relatively easy to run and interpret. Recent debate has noted the potential shortcomings of univariate approaches (Karnath *et al.*, 2018) including the inability to detect conditional voxel combinations (DeMarco and Turkeltaub, 2018) and mislocalization (Mah *et al.*, 2014), which might be addressed by multivariate analyses (but see Sperber *et al.*, 2019). The power of multivariate analyses, however, bring new interpretation challenges that are straightforward in univariate approaches: because all weights in multivariate models are conditional on each other, the interpretation or *post hoc* thresholding of individual weights becomes non-trivial (Haufe *et al.*, 2014). Accordingly, making inferences about local brain-behaviour relationships based on multivariate models is, at best, complicated. One transparent way forward is for studies to begin to present both univariate and multivariate results. Therefore, in the current study we show the results for four different methodological approaches, which allows us to demonstrate some commonalities and differences.

To extend our understanding of stroke aphasia to potentially critical aspects of non-verbal cognitive function and their structural correlates, we administered a comprehensive battery of non-verbal tests of attention and executive function to a large and diverse group of individuals with chronic post-stroke aphasia. The key aims of the study were: (i) to assess the prevalence of attention and executive dysfunction in patients with post-stroke aphasia; (ii) to explore the underlying relationships between the tests of attention and executive function, as well as the link to the patients’ language profiles; and (iii) to map the structural correlates for these underlying attention, executive and language features by means of four different methodological approaches.

## Materials and methods

### Participants

**Figure 1:**
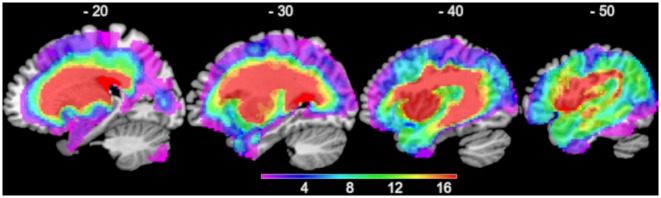
Overlap of the 38 patients’ lesions.

Thirty-eight participants were recruited for the present study (11 female, 27 male; mean age 64 ± 11.9 years, range 45–88 years; see Supplementary Table 1 for more details). All participants had a single left hemispheric stroke (ischaemic or haemorrhagic) at least 1 year before assessment and imaging (see Fig. 1 for lesion overlap map) and had no additional significant neurological conditions and no contraindications for MRI. They were pre-morbidly right-handed native English speakers with normal or corrected-to-normal vision. All had been diagnosed with aphasia but no restrictions were applied regarding the type of aphasia or the severity. Five patients are identical to patients whose data were reported in Halai *et al.* (2017) and Butler *et al.* (2014). Informed consent was obtained from all participants prior to participation, in line with the Declaration of Helsinki and as approved by the local NHS ethics committee. MRI data from a healthy age and education matched control group (10 female, 12 male) was used as a reference to identify lesion/abnormal tissue for each patient (Seghier *et al.*, 2008).

### Neuropsychological assessments

In addition to comprehensive language testing, described in more detail in Butler *et al.* (2014) and Halai *et al.* (2017), a broad range of standardized neuropsychological tests of attention and executive functions were administered. This included the subtests Alertness, GoNoGo, Divided Attention, and Distractibility from the Test of Attentional Performance (TAP Mobility version 1.3.1; Zimmermann and Fimm, 1995; www.psytest.net), a computerized test battery measuring reaction times and error rates in tests with varying attentional demands; the subtests Design Fluency and Trail Making (parts 2–4) from the Delis-Kaplan Executive Function System (D-KEFS; Delis *et al.*, 2001), the former assessing non-verbal idea generation by requiring participants to draw as many different figures as possible (connecting dots with lines), and the latter assessing visuospatial attention, processing speed and flexibility by requiring participants to connect numbers (part 2), letters (part 3) or alternatingly both (part 4) in ascending order; a computerized version of the Tower of London (TOL-F by Schuhfried; Kaller *et al.*, 2011), a visuospatial planning task; the Kramer test (Balzer *et al.*, 2011), a categorization task requiring participants to find ways of sorting eight cards into two groups; the Raven’s Coloured Progressive Matrices (Raven, 1962), assessing reasoning abilities; and the Brixton test (Burgess and Shallice, 1997), assessing visuospatial rule detection. Test scores were compared to published norms; age- and/or education-corrected norms were considered if available. For the Raven Matrices, the norms for part B were taken from Smits *et al. *(1997). Following Brooks *et al.* (2011), performance was considered as at least mildly-to-moderately impaired if it was more than 1.5 standard deviations (SD) below the mean (i.e. a T-score < 35, a percentile rank < 6 or a scaled score of ≤ 5).

### Data analysis

For a descriptive comparison of the impairments per patient and measure, and to account for missing data, percentages of impaired scores were calculated based on 16 measures from the 10 non-verbal tests and 14 measures from 12 language tests. The percentage of impaired scores per patient was taken as an indicator of the severity of their impairment and subsequently used in correlation analyses. Based on the raw test scores, three PCAs (correlation-based) were performed (using IBM SPSS 22.0) to elucidate the data’s underlying structure. The first PCA comprised just the non-verbal tests of attention and executive function. In the second PCA, only the language measures were included, which also provided a replication of previous results (Butler *et al.*, 2014; Halai *et al.*, 2017). Lastly, the third PCA comprised the combination of all measures included in the two other PCAs. To facilitate interpretation, it was ensured that a higher score would indicate better performance for all measures. To this end, reaction time measures were inverted, and accuracy rates were computed. Because of missing values and to include the same sample in all analyses, data of 32 of 38 patients were entered in the PCAs. TAP Distractibility and the letter and switching versions of the Trail Making Test were not included in order to not decrease the sample size further. Importantly, analyses including these measures showed that they were highly correlated with measures of the GoNoGo test or the number version of the Trail Making Test, respectively. To reduce the number of variables entered in the analysis, some comparable language measures were combined (Boston naming and Cambridge naming, immediate and delayed repetition of words and non-words, spoken and written word-picture matching, word and non-word minimal pairs). All components with eigenvalues ≥ 1 were extracted and then varimax rotated, yielding orthogonal and interpretable components. Two control analyses were performed to assess the stability and predictability of the PCA results. First, means and 95% confidence intervals for the component loadings were computed by leaving one case out each time. Second, the similarity between the observed data and those predicted was determined using a leave one case out method (by projecting the left-out case into the component space using the coefficient matrix). Correlations were computed to explore the relationship between component scores and the severity of the impairment in the neuropsychological tests as well as with patient characteristics such as lesion volume, age, and years of education.

### Neuroimaging data acquisition and analysis

High resolution structural T_1_-weighted MRI scans were acquired on a 3.0 T Philips Achieva scanner (Philips Healthcare) using an 8-element SENSE head coil. A T_1_-weighted inversion recovery sequence with 3D acquisition was used with the following parameters: repetition time = 9.0 ms, echo time = 3.93 ms, flip angle = 8°, 150 contiguous slices, slice thickness = 1 mm, acquired voxel size 1.0 × 1.0 × 1.0 mm, matrix size 256 × 256, field of view = 256 mm, inversion time = 1150 ms, SENSE acceleration factor 2.5, total scan acquisition time = 575 s.

Structural MRI scans were preprocessed with Statistical Parametric Mapping software (SPM8: Wellcome Trust Centre for Neuroimaging, http://www.fil.ion.ucl.ac.uk/spm/). The images were normalized into standard Montreal Neurological Institute (MNI) space using a modified unified segmentation-normalization procedure optimized for focal lesioned brains (Seghier *et al.*, 2008). Data from all participants with stroke aphasia and all healthy controls were entered into the segmentation-normalization. Images were then smoothed with an 8 mm full-width at half-maximum (FWHM) Gaussian kernel and used in the lesion analyses described below. An age and education matched healthy control group was used to determine the extent of abnormality per voxel. This was achieved using a fuzzy clustering fixed prototypes (FCP) approach, which measures the similarity between a voxel in the patient data with the mean of the same voxel in the control data (note: this method does not discriminate what caused the abnormality, but simply reflects how deviant the signal in the patient scan is from a healthy group). One can apply a threshold to the FCP to determine membership to abnormal/normal voxel. The default parameters were used apart from the lesion definition ‘U-threshold’, which was set to 0.5 to create a binary lesion image. We modified the U-threshold from 0.3 to 0.5 after comparing the results obtained from a sample of patients to what would be nominated as lesioned tissue by an expert neurologist. The images generated for each patient were visually inspected and manually corrected if necessary and were then used to create the lesion overlap map in Fig. 1.

The smoothed FCP images (% abnormality) were used to determine the brain regions where abnormality correlated with PCA component scores using a voxel-based correlational methodology (VBCM) (Tyler *et al.*, 2005), a variant of voxel-lesion symptom mapping (Bates *et al.*, 2003), in which both the behaviour and signal intensity measures are treated as continuous variables (conducted in SPM12). For the structural correlate analysis, we assume a negative correlation between abnormality and behavioural component score (i.e. greater abnormality leads to poorer performance). The participants’ component scores from the combined PCA, were entered simultaneously into a VBCM analysis. The resulting clusters thus account for the unique variance of a component. In additional analyses, lesion volume (calculated from the lesion identified by the automated lesion identification method; Seghier *et al.*, 2008), age, education, and time post-stroke were entered as covariates. Unless noted otherwise, we applied the threshold at voxel-level *P < *0.001 and family-wise error corrected (FWEc) cluster-level *P < *0.05.

To supplement the univariate analysis, we conducted multivariate analyses in two ways. First, we used the support-vector regression lesion symptom mapping (SVR-LSM) toolbox recently updated by DeMarco and Turkeltaub (2018), which is based on Zhang *et al.* (2014). In this framework, we loaded the lesion binary images as the features and created a separate model for each component score. The following settings were used: MATLAB SVM implementation, hyper-parameter optimization (Bayes optimization with default settings) and lesion threshold = 3 (∼10% of sample). The resulting beta weights were evaluated by permutation testing (*n* = 10 000, voxel-wise *P < *0.005 and cluster-wise *P < *0.05), but note that the model performance (predicted versus observed scores) is not evaluated in this approach. We ran two models per component, with and without correction for lesion volume (‘regress on both’). Second, we used the pattern recognition of neuroimaging toolbox (PRoNTo V2.1) (http://www.mlnl.cs.ucl.ac.uk/pronto/) (Schrouff *et al.*, 2013) as an alternative method because (i) it formally evaluates model predictions; and (ii) it does not truncate beta weights *post hoc.* For this toolkit, we entered the FCP % abnormality images as a continuous measure and followed the pipeline through in two pathways: (i) using the whole brain as input (similar to the VBCM); and (ii) restricted to lesion territory (*n* > 3) (similar to VLSM/SVR-LSM). Given the simplicity of the toolkit, we ran models using four regression machine implementations: (i) kernel ridge regression (KRR; Hastie *et al.*, 2009); (ii) relevance vector regression (RVR; Tipping, 2001); (iii) Gaussian processes regression (GPR; Rasmussen and Williams, 2006); and (iv) multi-kernel regression (MKR; Bach *et al.*, 2004; Rakotomamonjy *et al.*, 2008). PRoNTo relies on kernel methods to overcome the high dimensionality problem in neuroimaging (using *n* × *n* pair-wise similarity matrix) and features were mean centred. The default parameters were used for all machines and where necessary hyper-parameter optimization was achieved using nested leave-one-out cross validation (default grid search). A leave-one-out cross-validation scheme was used to determine model performance. For model inference, we report *P*-values for correlation and mean square error (MSE) following a permutation test of the observed scores (*n* = 1000) with a *P < *0.05 alpha threshold. As with the SVR-LSM, we ran each component model with and without lesion volume as a covariate.

The anatomical labels for the clusters were determined using the Harvard-Oxford atlas for grey matter and on the John Hopkins white matter atlas for white matter tracts. Furthermore, comparisons to existing findings were made by either overlapping the respective maps, if available, or by checking (in MRIcron) whether published peak coordinates overlapped with the clusters from the VBCM.

### Data availability

Behavioural data are available in the Supplementary material. Further data are potentially available by request to M.A.L-R.

## Results

### Neuropsychological profiles

**Figure 2:**
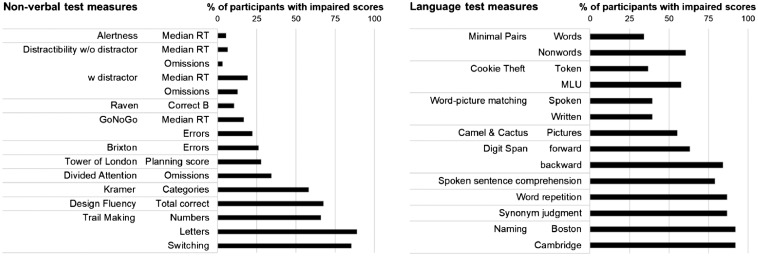
Percentage of participants with impaired performance on each measure of the non-verbal tests (*left*) and language tests (*right*).

**Figure 3:**
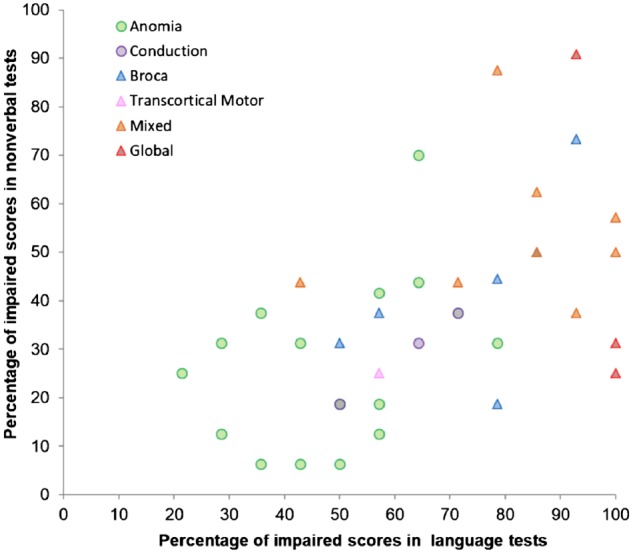
**Patients’ overall impairment in the non-verbal versus language tests.** The percentages of impaired scores correlated significantly (r_s_ = 0.591, *P < *0.01, *n* = 38, also if patient characteristics were accounted for by means of partial correlations). Symbols and colours denote an individual’s aphasia type based on the BDAE (triangles for non-fluent, circles for fluent patients, for colours see *top left* legend). More saturated or differently coloured symbols denote two patients in the same spot.

The first aim of this study was to assess the prevalence of impairments in attention and executive functions in patients with post-stroke aphasia. Patients’ performance was thus compared to available norm data to identify the number of impaired scores per patient and test. All participants scored below normal range in at least one measure of the 10 tests of attention and executive function, but no participant was impaired in all of these tests (mean percentage of impaired scores per patient 36.7 ± 20.8%, range 6.3–90.9%). Fifteen patients were impaired in at least half of the administered non-verbal tests. In comparison to the non-verbal test performance, all participants scored below normal range in at least three measures of the 12 language tests, 30 patients were impaired in at least half of the administered language tests, and five participants were impaired in all of these tests (mean percentage of impaired scores per patient 65.0 ± 22.4%, range 21.4–100%). Details on impaired performance in the non-verbal and language tests are depicted in Fig. 2, while Fig. 3 shows the patients’ overall impairment in the non-verbal versus language tests (as percentage of impaired scores in the respective tests). Individual patients’ scores are available in Supplementary Tables 2 and 3, while Supplementary Fig. 2 gives details about impaired performance on the different principal components.

The Alertness test and the Distractibility without distractor condition were the only two non-verbal tests where the percentage of impaired scores was around or below 5% of the sample. These tests measure more basic attention functions and it has previously been reported that these aspects of attention are more commonly impaired in right-hemispheric stroke patients (Sturm *et al.*, 1997). The tests with the highest percentages of impaired scores were the Trail Making Test [numbers impaired in 25 patients (65.8%), letters in 32 patients (88.9%), and switching in 29 patients (85.3%)], the Design Fluency Test (25 patients, 67.6%) and the Kramer Test (21 patients, 58.3%). We split the sample into two groups of ‘cognitive’ severity based on a median split of overall impairment in the non-verbal tests (see Supplementary Table 2 for details). Comparison of the two groups revealed that only the more cognitively-severe patients had impaired scores in the Tower of London and TAP Divided Attention tests. As such, the test of divided attention might be especially clinically useful as a predictor of impaired cognition in aphasic populations. In contrast, both groups showed a similar and high degree of impairment in two other tests, the Kramer and the letter version of the Trail Making Test. The high percentage of impaired performance in the Trail Making Test is particularly important considering the widespread use of this test with aphasic patients. Thus, impaired performance in the switching condition of the trail making test need not necessarily stem from difficulties in switching but from reduced automaticity of accessing the letters in order (and, to a lesser extent, numbers), which is a prerequisite for task completion.

### Separate and combined principal component analyses of non-verbal and language tests

**Figure 4:**
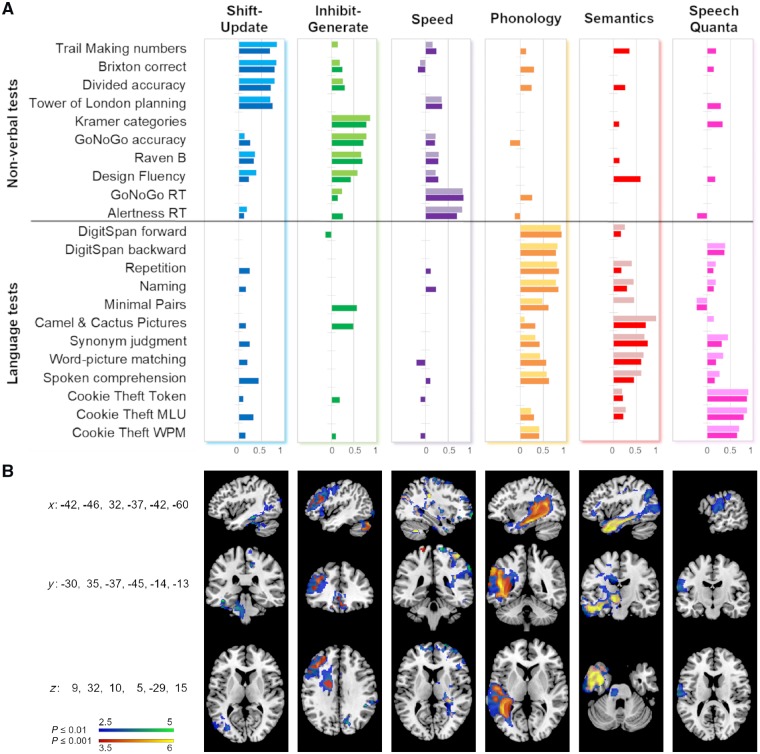
**Component loadings and structural correlates associated with each component. **(**A**) The darker coloured bars (from *left* to *right*: blue, green, purple, orange, red, pink) represent the loadings on the six components from the combined PCA. The lighter coloured bars represent the loadings on the three components in the separate non-verbal-only PCA (first three columns) and the language-only PCA (last three columns). Loadings < 0.1 are not depicted. MLU = mean length of utterance; WPM = words per minute. (**B**) Structural correlates associated with each component from the combined PCA. Clusters shown in blue-green were obtained by applying a voxel-level threshold of *P ≤ *0.01, clusters in red-yellow correspond to a voxel-level threshold of *P ≤ *0.001. A family-wise error correction of *P ≤ *0.05 was applied to all clusters. The respective coordinates in MNI-space are indicated on the left side. Figures are in neurological convention (left is left).

The second aim was to explore the underlying relationships between the tests of attention and executive function, as well as linking these to the patients’ language profiles. We computed separate PCAs for the non-verbal and verbal tests, as well as a combined PCA including all tests. The PCA including only the non-verbal tests of attention and executive functions yielded three orthogonal components accounting for 68.5% of the variance [Kaiser-Meyer-Olkin (KMO) = 0.704]. Based on the tests loading highest on each component (Fig. 4A), the first component (accounting for 28.1% of the variance) was interpreted as ‘shift-update’ as the tests loading highest are relatively demanding with respect to flexible (visuo-spatial) processing and working memory. Interestingly, the first component contains tests that are traditionally regarded as tests of executive function (Tower of London, Brixton) as well as tests that are more associated with attention (Divided attention and Trails numbers), which underlines the link between the two domains that is also reflected in the term ‘executive attention’ (Kane and Engle, 2002; Petersen and Posner, 2012). The second component (23.2%) was interpreted as ‘inhibit-generate’ as it included tests like the Kramer sorting test (requiring idea generation as well as inhibition of salient aspects of the stimuli) as well as simple response inhibition tasks like the GoNoGo test. The third component (17.2%) was interpreted as ‘speed’ as it contained the reaction time measures of both basic attention tasks.

The separate analysis of the language tests yielded three orthogonal components accounting for 78.3% of the variance (KMO = 0.718). The components can be interpreted as ‘phonology’ (accounting for 31.5% of the variance), ‘semantics’ (24.2%), and ‘speech quanta’ (22.6%), directly replicating previous research (Butler *et al.*, 2014; Halai *et al.*, 2017). The fact that the patient sample of this study largely consists of patients not included in previous reports shows the stability of these results. Moreover, other groups report similar patterns (Mirman *et al.*, 2015; Lacey *et al.*, 2017).

The third PCA—combining the non-verbal and language tests—yielded six orthogonal components accounting for 78.6% of the variance (KMO = 0.661). Figure 4A shows that the components from the two separate analyses remained relatively stable (also evidenced by high correlations between the separate and combined component scores; Table 1 and Supplementary Fig. 2). Their order and percentage of explained variance was as follows: phonology (21.6%), shift-update (13.4%), inhibit-generate (12.2%), speech quanta (11.7%), semantics (11.5%), speed (8.2%). Notably, apart from the phonology component which explained the highest amount of variance, the other language and non-verbal components are weighted similarly in terms of explained variance.

**Table 1:** Spearman correlations within and between severity of non-verbal and language impairment, component scores, and patient characteristics

	Severity		Non-verbal PCA			Patient characteristics			
	Non-verbal	Verbal	S-U	I-G	Speed	Lesion	Age	Education	Time post-stroke
**Severity**									
Verbal	0.535*	–	−0.521*	−0.105	0.150	–	–	–	–
Non-verbal	–	–	**−0.676** *	−0.283	−0.110	–	–	–	–
**Verbal PCA**	–	–							
Phonology	−0.216	**−0.719** *	0.261	−0.294	0.025	−0.208	−0.126	−0.209	0.131
Semantics	−0.316	−0.383*	0.421*	0.373*	0.087	−0.396*	−0.433*	0.288	−0.115
Speech Quanta	−0.362*	−0.427*	0.443*	0.097	−0.283	−0.504*	0.068	0.190	−0.240
**Combined PCA**									
Phonology	−0.164	**−0.744** *	0.216	−0.245	−0.062	−0.238	−0.121	−0.213	0.126
S-U	−0.530*	−0.259	0.871*	−0.063	−0.018	−0.308	−0.445*	0.143	−0.393*
I-G	−0.235	−0.173	−0.109	0.905*	−0.184	0.050	−0.436*	0.312	−0.208
Speech Quanta	−0.325	−0.349	0.214	0.178	−0.195	−0.376*	0.053	0.120	−0.142
Semantics	−0.139	−0.118	0.194	0.201	−0.010	−0.370*	−0.014	0.247	0.061
Speed	−0.172	0.103	−0.122	0.037	0.902*	0.177	−0.305	−0.003	0.294
**Patient characteristics**									
Time post-stroke	0.196	0.151	−0.381*	−0.187	0.240	0.389*	0.094	−0.123	–
Education	−0.279	−0.061	0.254	0.494*	−0.174	−0.132	−0.321	–	–
Age	0.323	0.332	−0.441*	−0.455*	−0.254	0.251	–	–	–
Lesion	0.353*	0.555*	−0.518*	−0.146	0.156	–	–	–	–

S-U = shift-update; I-G = inhibit-generate.

*P < 0.05 two-tailed; bold = significant after Bonferroni correction (*P* < 0.0004); *n* = 32.

The stability analyses for all three PCAs revealed that all test loadings had very tight 95% confidence intervals. The most unstable tests were Design Fluency in the non-verbal PCA (mean loading = 0.58 ± 0.02), Camel and Cactus in the verbal PCA (0.86 ± 0.08), and Kramer in the combined PCA (0.75 ± 0.05). We also found generally high correlations between the predicted left-out cases and observed scores for the non-verbal (r = 0.83), verbal (r = 0.88) and combined (r = 0.88) PCAs.

Whilst the combined PCA preserves the nature of the six principal behavioural components, it is notable that many individual language tasks load across verbal and non-verbal components, reflecting the fact that many language activities and the tasks used to assess them require generalized attention and executive skills (e.g. comparing verbal stimuli, deciding between responses, etc.). This is true for both semantic tests (aligning with the fact that semantic cognition requires both access to semantic representation but also executively-related processes (Jefferies and Lambon Ralph, 2006; Thompson *et al.*, 2018) and for phonological tests with demands on working memory (sentence comprehension) or abstract reasoning and problem-solving (minimal pairs).

### Relationship between impairment, component scores, and patient characteristics

Previous research documents both the presence (Fucetola *et al.*, 2009; Baldo *et al.*, 2015) and absence (Helm-Estabrooks, 2002) of a significant correlation between non-verbal and verbal impairment. We found a moderate but significant relationship between simple indices of non-verbal and language impairment (in terms of percentage of impaired non-verbal/language test scores per patient), as shown in Fig. 3 and Table 1. This finding seems to relate primarily to the non-verbal shift-update component that correlates with both indices of severity. Beyond this, there is considerable variation, which results from the fact that even when combined into one PCA there are statistically-orthogonal components for the language and non-verbal test scores; they would collapse into a shared PCA component if performance in non-verbal and language tests was a reflection of simple severity alone.

Regarding patient characteristics, also shown in Table 1, non-verbal as well as verbal severity correlated significantly with lesion volume, but neither correlated with age, education or time post-stroke. More specifically, lesion volume correlated with the separate non-verbal shift-update component and with the semantic and speech quanta components of both PCAs. Age correlated with the non-verbal components apart from speed, and with the semantic component from the separate verbal PCA. Education only correlated significantly with the inhibit-generate component from the separate non-verbal PCA, and time post-stroke correlated moderately with the shift-update components.

Notably, the first non-verbal and language components, shift-update and phonology, were still significantly correlated with the severity of the non-verbal and language impairment, respectively, when age, education, time post-stroke and lesion volume were accounted for by means of partial correlation (separate shift-update component and non-verbal impairment r = − 0.629; separate/combined phonology component and language impairment r = −0.814/r = −0.851; all *P < *0.0004).

### Structural correlates

**Figure 5:**
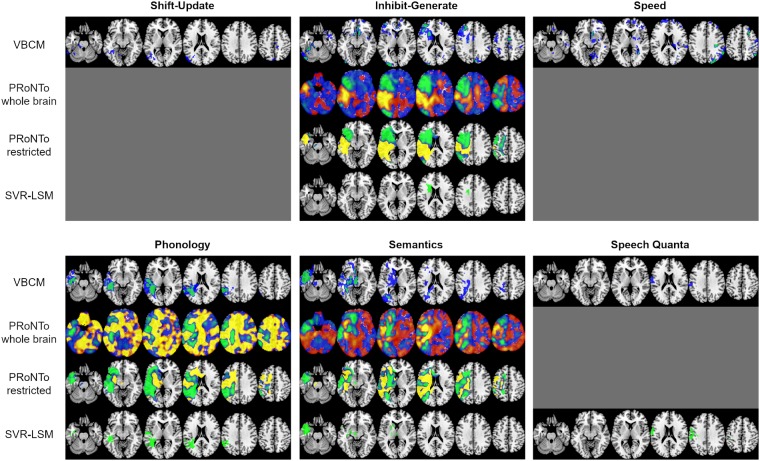
**Comparison of brain-behaviour mapping results based on the four different methodological approaches. **The significant VBCM clusters are shown in blue (voxel-level threshold 0.01) and green (voxel-level threshold 0.001), a family-wise error correction of *P ≤ *0.05 was applied to all clusters, and images are thresholded at the respective minimum t-value. The PRoNTo results depict the weights for the winning model if significant (see text), either including the whole brain space or restricting it to lesion territory (*n* > 3). They are thresholded from −0.005 to −0.0001 (green-blue) and 0.0001 to 0.005 (red-yellow). The negative weights are considered as more meaningful in this approach. The SVR-LSM images show voxels with significant beta weights after permutation testing (*n* = 10 000, voxel-wise *P < *0.005 and cluster-wise *P < *0.05). MNI coordinates of slices, from *left* to *right*, are z = −25, −10, 5, 20, 35, 50 and they are in neurological convention (left is left). A grey surface indicates that no significant results were found for the respective component and methodological approach.

The third aim was to map the structural correlates for the underlying attention, executive and language features. We simultaneously entered all component scores obtained in the combined PCA and performed a VBCM with tissue abnormality, which yielded significant clusters for all components (though shift-update and speech quanta were present at a lower voxel-level threshold of 0.01, FWEc at cluster-level *P < *0.05). The clusters are depicted in Figs 4B and 5, and details are listed in Table 2.

**Table 2:** Clusters and peaks associated with the non-verbal and language components

Component	Extent	Location	Left/right	Z	*x*	*y*	*z*
**Shift-Update**	2032	Temporal fusiform cortex posterior	Left	4.29	−40	−32	−16
		Temporal fusiform cortex posterior	Left	3.69	−38	−34	−30
		Inferior longitudinal fasciculus	Left	3.58	−42	−36	−14
		Temporal fusiform cortex posterior	Left	3.32	−42	−30	−28
		Inferior temporal gyrus temporo-occipital	Left	3.27	−60	−56	−22
		Occipital fusiform gyrus	Left	3.22	−26	−64	−16
		Lateral occipital cortex superior	Left	3.22	−56	−72	20
		Inferior temporal gyrus temporo-occipital	Left	3.19	−56	−50	−22
	990	Left Precuneous cortex	Left	4.04	−2	−62	66
		Postcentral gyrus	Right	3.78	10	−36	72
		Precentral gyrus	Right	3.76	10	−32	50
		Corticospinal tract	Right	3.64	16	−34	54
		Superior parietal lobule	Right	3.57	10	−48	72
**Inhibit-Generate**	1270	Frontal pole	Left	5.00	−20	56	12
		Frontal pole	Left	3.99	−28	50	16
		Middle frontal gyrus	Left	3.94	−38	28	32
		Frontal pole	Left	3.91	−28	42	36
		Frontal pole	Left	3.63	−38	52	0
		Middle frontal gyrus	Left	3.60	−44	24	24
		Inferior frontal gyrus pars triangularis	Left	3.50	−40	32	18
		Middle frontal gyrus	Left	3.45	−52	18	30
	530	Subcallosal cortex	Right	5.06	6	26	−14
		Accumbens	Right	4.95	8	16	−6
		Cingulate gyrus anterior	Right	4.01	2	36	2
		Accumbens	Left	3.88	−8	12	−8
		Subcallosal cortex	Left	3.84	−12	28	−16
	447	Occipital pole	Left	4.27	−24	−96	16
		Occipital pole	Left	4.21	−22	−94	10
		Lateral occipital cortex inferior	Left	3.75	−42	−88	−10
	414	Supplementary motor cortex	Left	3.55	−16	−10	34
		Anterior thalamic radiation	Left	3.44	−20	20	18
		Superior longitudinal fasciculus	Left	3.34	−22	−4	30
	337	Supplementary motor cortex	Right	4.50	6	−12	46
		Cingulate gyrus posterior	Right	4.42	4	−22	42
**Speed**	369	Lateral occipital cortex superior	Right	4.51	26	−86	34
		Occipital pole	Right	4.50	22	−90	32
	355	Angular gyrus	Right	4.53	62	−54	38
		Lateral occipital cortex superior	Right	4.12	54	−62	28
**Phonology**	5688	Inferior longitudinal fasciculus	Left	5.99	−42	−30	−16
		Inferior longitudinal fasciculus	Left	5.70	−42	−34	−14
		Temporal fusiform cortex posterior	Left	5.58	−40	−24	−18
		Inferior temporal gyrus posterior	Left	5.11	−50	−18	−24
		Inferior temporal gyrus temporo-occipital	Left	4.93	−48	−46	12
		Supramarginal gyrus posterior	Left	4.61	−60	−48	34
		Angular gyrus	Left	4.56	−40	−54	14
		Middle temporal gyrus temporo-occipital	Left	4.47	−42	−54	8
		Planum temporale	Left	4.37	−36	−32	14
**Semantics**	4994	Temporal fusiform cortex posterior	Left	5.48	−40	−30	−16
		Inferior temporal gyrus posterior	Left	5.15	−52	−16	−24
		Parahippocampal gyrus anterior	Left	5.12	−34	−6	−26
		Thalamus	Left	5.12	−10	−22	−4
		Temporal Pole	Left	5.04	−52	10	−36
		Hippocampus	Left	5.02	−34	−10	−24
		Anterior thalamic radiation	Left	4.90	−10	−18	−8
		Anterior thalamic radiation	Left	4.82	−8	−18	−12
		Inferior longitudinal fasciculus	Left	4.71	−40	−36	−14
**Speech Quanta**	1010	Postcentral gyrus	Left	3.24	−66	−16	16
		Postcentral gyrus	Left	2.80	−56	−12	28
		Supramarginal gyrus anterior	Left	2.63	−62	−28	36
		Postcentral gyrus	Left	2.51	−50	−24	38
		Postcentral gyrus	Left	2.50	−44	−24	44
		Precentral gyrus	Left	2.37	−60	0	38

Only clusters with cluster-level FWEc *P* ≤ 0.001 are shown in the table.

From the non-verbal components, shift-update was uniquely correlated with left lateral temporo-occipital regions (encompassing parts of the medial and inferior temporal gyrus, fusiform cortex as well as the lateral occipital cortex and extending to parahippocampal regions and brain stem), in addition to bilateral mainly parietal midline regions (postcentral gyrus, precuneus, superior parietal lobule). The inhibit-generate component was uniquely correlated with left lateral (middle and inferior frontal gyrus) and subcortical frontal regions (anterior thalamic radiation) as well as medial frontal regions bilaterally (subcallosal cortex, (para)cingulate gyrus, supplementary motor cortex), in addition to several smaller clusters in occipital and parietal regions. The speed component was also associated with several small, mainly right-sided parieto-occipital and frontal clusters.

The clusters associated with the three language components resembled the clusters reported in previous studies by our group (Butler *et al.*, 2014; Halai *et al.*, 2017). The phonology cluster was uniquely correlated with left temporo-parietal regions encompassing parts of the inferior, middle, and superior temporal gyri as well as supramarginal and angular gyrus. The semantics component was associated with a cluster of left cortical (anterior temporal lobe, extending inferiorly into occipital lobe) and subcortical (thalamus) regions. The speech quanta cluster was in the dorsal fronto-parietal cortex and included parts of the pre- and postcentral gyrus. When lesion volume was included as a covariate, inhibit-generate, speed, and phonology remained significant. Semantics was only significant at a less strict threshold; this applied as well to the shift-update component and is shown in Supplementary Fig. 3. The effects of including other patient characteristics such as age, education, and time post-stroke in the VBCM are also shown and discussed in the Supplementary material.

The multivariate analyses yielded similar results, as shown in Fig. 5. The SVR-LSM produced significant clusters for inhibit-generate, phonology, semantics and speech quanta. The evaluation of the best model within PRoNTo revealed significant brain-behaviour relationships for inhibit-generate (KRR model cross-validation r = 0.357, MSE = 0.854, *P = *0.022), phonology (MKR model cross-validation r = 0.379, MSE = 1.008, *P = *0.042), and semantics (KRR model cross-validation r = 0.750, MSE = 0.431, *P < *0. 001) when using the whole brain. The results were the same when using the restricted lesion territory: inhibit-generate (KRR model cross-validation r = 0.400, MSE = 0.816, *P = *0.019), phonology (GPR model cross-validation r = 0.359, MSE = 0.860, *P = *0.013), and semantics (KRR model cross-validation r = 0.712, MSE = 0.478, *P < *0.001). When lesion volume was added as a covariate, the SVR-LSM produced significant clusters for inhibit-generate and phonology only, while the PRoNTo toolkit found significant models for inhibit-generate and semantics (for both whole brain and restricted lesion territory), as detailed in Supplementary material.

As can be seen in Fig. 5, the VBCM and SVR-LSM results were strikingly similar. For inhibit-generate, VBCM yielded bigger and more distributed clusters but there was an overlap with the significant SVR-LSM result in left frontal subcortical regions. For phonology, the SVR-LSM and VBCM clusters were nearly identical, with the former extending slightly more into the superior parietal cortex, and the latter extending more anteriorly in the temporal lobe. Likewise, the VBCM and SVR-LSM results for the semantics component overlapped largely, with the former being slightly bigger and extending further posteriorly in the ventral temporal lobe. Finally, the main difference regarding the speech quanta results was that the SVR-LSM cluster extended slightly more dorsally and anteriorly. Furthermore, the unthresholded beta maps from PRoNTo showed some correspondence to both VBCM and SVR-LSM in terms of the negative beta weights. Apart from a small set of voxels in the medial temporal lobe that was part of the VBCM semantics cluster, all voxels identified in the VBCM and SVR-LSM analyses were within regions that were given a (strong) negative weight in the PRoNTo models. In contrast to SVR-LSM, the PRoNTo beta maps show the weights of the entire input space after confirming the model significantly maps to behaviour.

## Discussion

Even though there is growing awareness of the importance of attentional and executive (dys)functions in aphasia, to date the occurrence and patterns of such impairments, the relationship between non-verbal and language functions, as well as their structural correlates have not been studied in detail in the same sample of patients. This study extended our understanding of the multidimensionality of chronic post-stroke aphasia and found that: (i) a considerable number of patients showed impaired performance in tests of attention and executive function; (ii) the variance underlying non-verbal and language test performance was best captured by three orthogonal components each; and (iii) both univariate and multivariate mapping approaches revealed brain-behaviour relationships in line with previous studies based on other methodologies and populations.

Given that our sample consisted of patients diagnosed with aphasia, unsurprisingly the incidence of language impairments was high and performance in language tests was overall worse than in non-verbal tests. However, patients’ performance in tests of attention and executive function was also considerably impaired, as none of the patients performed within normal range in all tests and nearly 50% of the patients showed deficits in at least half of the administered tests. While language impairments might be the most salient consequences of a left hemispheric stroke, our more thorough and systematic investigation replicates earlier observations of co-occurring deficits in other cognitive domains (Helm-Estabrooks, 2002; Murray, 2012; Marinelli *et al.*, 2017; Ramsey *et al.*, 2017); a pattern that is important for clinical management and response to rehabilitation.

Our comprehensive battery of non-verbal tests allowed us to identify three separable components of attention and executive function (shift-update, inhibit-generate, and speed), which mirror explorations in healthy participants (Petersen and Posner, 2012; Friedman and Miyake, 2017). This contrasts with current studies in aphasia and clinical practice that either fail to assess non-verbal functions at all, or if they do then only a few (screening) measures are used. Whilst there are clear co-occurrences and simple raw correlations between measures, there is little evidence that everything collapses to one simple severity-based metric. This is in line with a recent study by Marinelli *et al.* (2017), reporting that only a quarter of their severely aphasic patients was also severely impaired in non-verbal cognition, as well as classical findings showing that language and non-language performance in aphasia have low correlations, and that aphasia cannot be reduced to simple cognitive severity (Basso *et al.*, 1973; Helm-Estabrooks, 2002; Fucetola *et al.*, 2009).

It is important to note that performance on the various components is independent, suggesting that patients have variable combinations of verbal and non-verbal deficits. The common co-occurrence is relevant for three main reasons: (i) many language assessments also load on attention and executive functions; (ii) some aspects of language function require interactions between components (e.g. controlled semantic processing: Jefferies and Lambon Ralph, 2006); (iii) response to therapy and recovery has been shown to relate not only to language severity but also to more domain-general functions (Lambon Ralph *et al.*, 2010; Geranmayeh *et al.*, 2017; Conroy *et al.*, 2018). Our findings thus imply that the three identified non-verbal cognitive components need to be assessed separately in future studies and in clinical practice, as they might have different implications for function and recovery. Likewise, interventions should be considered in this patient population that (i) specifically aim at improving domain-general cognitive deficits (Geranmayeh *et al.*, 2017); (ii) integrate therapy of attentional or executive dysfunctions into speech-language remediation (Mayer *et al.*, 2017); and (iii) adopt a multidisciplinary team approach.

Using univariate and multivariate brain-behaviour mapping approaches we identified separable structural correlates for all three non-verbal components, in addition to replicating previous findings regarding the structural correlates of the three verbal components. The clusters of all three non-verbal components overlapped to some degree with the multi-demand network (Duncan, 2010; Fedorenko *et al.*, 2013). In addition, the shift-update cluster overlapped with the dorsal attention and control network, while the inhibit-generate cluster overlapped with the ventral attention and control network (Yeo *et al.*, 2011). More specifically, the correlates of shift-update fit well with task-based functional imaging studies that report activations in lateral temporo-occipital areas for demanding visuo-spatial tasks (Fedorenko *et al.*, 2013; Humphreys and Lambon Ralph, 2017) or when location and feature information must be combined (Simpson *et al.*, 2011); both processes are inherent to shift-update. The findings for the inhibit-generate component are also in line with previous research. Although more extensive, this network of areas overlaps with the regions found in a previous study of aphasia (Lacey *et al.*, 2017) and those identified in a meta-analysis of functional imaging studies on executive functions (Niendam *et al.*, 2012).

From a methodological point of view, it is important to note the complementary differences between the interpretation of univariate and multivariate analyses (Hebart and Baker, 2018). In general, with univariate analyses, the beta values assigned to voxels are relatively transparent (i.e. their sign and strength indicates meaningful relationships with behaviour) and thus inferences about local function are easier to make (although inference using cluster-level thresholds can only show that there is signal somewhere in the cluster; Woo *et al.*, 2014). However, univariate methods are limited by practical (i.e. multiple comparison correction, interactions between multiple variables that are typically not orthogonal) and theoretical concerns (i.e. assumption of voxel independence, mislocalization of effects; Mah *et al.*, 2014; DeMarco and Turkeltaub, 2018; Karnath *et al.*, 2018). In contrast, multivariate methods can be used for encoding or decoding (Naselaris *et al.*, 2011; Hebart and Baker, 2018) and have different goals (i.e. to predict data from experimental conditions or to map brain status to behavioural performance and make formal predictions, respectively). These models can have problems with interpretability as feature weights become non-transparent (Haufe *et al.*, 2014; Hebart and Baker, 2018), although encoding can assist with this challenge to some degree (such as partial least squares and canonical correlation analysis). By definition, in multivariate analyses all voxel/feature weights are non-independent and thus the importance of these weights is not easy to interpret. Furthermore, analysis steps that select a subsample of weights automatically mean that the overall multivariate model has been changed and one would need to test (i) whether the contribution of a voxel to the model is greater than chance; or (ii) whether the contribution of a voxel to the model is stable across different samples (e.g. via bootstrapping; Kuceyeski *et al.*, 2016). Given these differences between the methods, it is striking that the multivariate models (both SVR-LSM and PRoNTo) produced beta maps that strongly correspond to the VBCM results. We assume this follows the fact that stroke tends to generate binary tissue status (intact versus infarcted) and this will dominate the predictions of behavioural variation in all models (and are the most likely features to be selected in any form of weight truncation such as that used in SVR-LSM). There are some potential avenues to help improve interpretations of both univariate and multivariate methods in the future. First, a recent study showed that it may be possible to compute a correction for the mislocalization caused by anatomical bias (Sperber and Karnath, 2017). Second, Haufe *et al.* (2014) and Naselaris *et al.* (2011) propose ways in which a decoding model can be transformed into an encoding model, which potentially leads to interpretable weights. Third, alternative sparse algorithms (such as LASSO, elastic net or recursive feature selection) have the benefit of introducing a penalty for complexity and therefore provide a solution with the smallest number of features (though the challenge of interpreting the resultant weights still holds). Finally, we note that multivariate decoding methodologies typically require a large dataset, as data are partitioned into training/test sets for cross validation. This can be practically challenging, as not only do we require neuroimaging data but also a large neuropsychological test battery to determine the underlying principal components. In a recent simulation study (Sperber *et al.*, 2019), it was suggested that ∼100 subjects are required to have stable/reproducible beta parameter mapping, whereas for prediction of clinical outcomes the number peaked at 40 and was relatively stable from this point up to 100 cases. In the current study we obtained 32 cases (similar to Lacey *et al.*, 2017) and so future work will require replication based on larger groups sizes.

Overall, the structural correlates align with areas of different cognitive functions in healthy participants. The variable combinations of verbal and non-verbal deficits observed across post-stroke aphasia (see above) presumably reflect differential encroachment of each person’s lesion on the various regions implicated for each non-verbal and verbal component and/or their connections. This would imply that interventions should target different brain regions depending on which component needs to be ameliorated to improve performance. Options to be explored include neurostimulation, for instance by targeting medial frontal areas (Sliwinska *et al.*, 2017) or pharmacology (Berthier *et al.*, 2011). It also has implications for building accurate prediction models (Price *et al.*, 2010; Hope *et al.*, 2013, 2018; Yourganov *et al.*, 2015, 2016; Pustina *et al.*, 2017; Thye and Mirman, 2018). First, it may be that predictions of language performance might be improved if the predictors include non-verbal cognitive abilities alongside patient characteristics. Second, it may be possible to improve prediction models of both verbal and non-verbal abilities by using these updated PCA-derived structural correlates (*cf.*Halai *et al.*, 2018).

In conclusion, this study was able to demonstrate that functionally distinct aspects of attention and executive skills are commonly impaired in patients with post-stroke aphasia. The assessments successfully used here could be adopted in clinical assessment to guide management and choices over clinical pathways. Furthermore, future investigations can explore which specific aspects of attention and executive function are crucial for effective therapy and good rehabilitation outcomes, and how these features of non-verbal abilities can be supported or boosted through novel interventions.

## Supplementary Material

awz258_Supplementary_Data

## Abbreviations

PCA: principal component analysis
PRoNTo: pattern recognition of neuroimaging toolbox
SVR-LSM: support-vector regression lesion symptom mapping
VBCM: voxel-based correlational methodology
