# Supplementary material for: Assessing and mapping language, attention and executive multidimensional deficits in stroke aphasia
Source: Brain. 2019 Sep 5;142(10):3202–16. doi: 10.1093/brain/awz258 (PMC6794940; doi:10.1093/brain/awz258)
Supplement: awz258_Supplementary_Data [file awz258_supplementary_data.pdf]

Assessing and mapping language, attention and executive multidimensional deficits in stroke aphasia

Rahel Schumacher, Ajay D. Halai, Matthew A. Lambon Ralph

SUPPLEMENTARY MATERIAL

Contents

Abbreviations 3

Supplementary Table 1..... 4

Supplementary Table 2..... 5

Supplementary Table 3..... 6

Supplementary Fig. 1 7

Supplementary Fig. 2 9

Supplementary Fig. 3 11

Supplementary Fig. 4 12

Supplementary Fig. 5 14

References 16

Abbreviations

BDAE = Boston Diagnostic Aphasia Examination

CCT = Camel & Cactus Test

MLU = Mean length of utterance

NW = Nonword

RT = Reaction Time

TMT = Trail Making Test

TOL = Tower of London

W = Word

WPM = Words per minute

Supplementary Table 1. Participant background information.

Subj	Age	Gender	Education	Years post-stroke	Lesion Volume	BDAE classification*
1	55	m	17	9	11915	Broca
2	55	f	12	12	9767	Anomia
3	71	m	11	8	8788	Mixed Nonfluent
4	61	m	11	17	18392	Broca
5	72	m	12	10	41379	Global
6	47	m	11	6	8437	Anomia
7	76	m	11	4	22732	Mixed Nonfluent
8	50	f	11	8	6975	Anomia
9	79	f	11	7	13577	Anomia
10	63	f	19	6	9159	Anomia
11	80	m	13	5	34242	Mixed Nonfluent
12	71	m	11	4	3311	Anomia
13	71	m	13	6	13080	Broca
14	62	m	11	5	16433	Anomia
15	70	m	13	7	33239	Global
16	52	m	13	8	22948	Anomia
17	48	f	16	4	3897	Conduction
18	84	m	10	3	12131	Broca
19	46	f	13	5	18948	Anomia
20	75	f	11	6	23863	TMA
21	76	f	11	15	12057	Mixed Nonfluent
22	45	f	16	3	175	Anomia
23	66	m	11	4	33239	Mixed Nonfluent
24	69	m	11	5	31317	Mixed Nonfluent
25	81	m	11	6	33678	Mixed Nonfluent
26	47	m	11	3	10409	Anomia
27	59	f	11	24	12699	Anomia
28	68	m	11	2	4879	Conduction
29	53	m	11	7	37822	Global
30	88	m	9	2	8528	Anomia
31	75	m	11	11	36877	Broca
32	67	m	17	2	6557	Conduction
33	57	m	16	2	6974	Anomia
34	66	m	10	7	6607	Anomia
35	50	m	19	2	4538	Anomia
36	51	m	11	3	14681	Anomia
37	56	f	11	2	10081	Mixed Nonfluent
38	69	m	12	7	37907	Broca/Mixed Nonfluent

* This information is provided for completeness only. Issues with these diagnostic categories are discussed elsewhere (e.g. Dronkers and Larsen, 2001; Butler *et al.*, 2014; Kasselimis *et al.*, 2017).

Supplementary Table 2. Participants' scores in tests of executive function and attention.

Subj	TMT			Brixton	Divided	TOL	Kramer	Raven B	Design Fluency	GoNoGo		Alertness	Distract w/o distractor		Distract w distractor	
	num	let	swi	err	omi	plan	cat	cor	cor	RT	err	RT	RT	omi	RT	omi
1	58	**82**	**161**	**27**	**6**	**6**	3	11	32	438	0	221	566	0	**624**	0
2	**74**	52	108	22	2	11	**2**	10	26	446	1	277	510	0	512	0
3	82	**121**	**277**	18	**14**	**6**	**0**	7	**14**	**569**	5	291	424	1	482	1
4	47	**163**	**309**	24	5	11	**2**	7	19	426	**9**	229	593	0	**639**	0
5	**a 500**	**a 500**	**a 500**	**34**	a	**0**	**0**	**1**	**4**	**737**	**11**	208	a	a	a	a
6	**62**	**95**	**182**	17	4	12	**2**	12	**17**	485	1	221	528	1	538	0
7	**540**	**a 240**	n/ad	23	n/ad	**0**	**0**	**3**	**3**	a	a	**1223**	n/ad	n/ad	n/ad	n/ad
8	**102**	**142**	**393**	**31**	**17**	8	4	10	**15**	407	**7**	211	482	0	517	1
9	**214**	n/ad	n/ad	**38**	**9**	**0**	**0**	6	**11**	525	**14**	234	n/ad	n/ad	n/ad	n/ad
10	44	**85**	118	19	2	13	5	12	24	452	1	256	506	0	520	0
11	**194**	**220**	**372**	22	**18**	6	**1**	9	**9**	557	3	324	586	0	**647**	1
12	51	76	**203**	19	1	11	**1**	5	**14**	478	3	207	502	0	524	0
13	59	**104**	**136**	**31**	n/av	15	n/av	10	n/av	n/av	n/av	n/av	n/av	n/av	n/av	n/av
14	**150**	**151**	**220**	12	4	14	3	11	24	527	0	194	513	0	463	0
15	**114**	**129**	**384**	28	2	10	**0**	10	**15**	484	4	214	426	0	442	0
16	52	**125**	**378**	20	2	16	**2**	9	**10**	462	**6**	286	519	0	513	0
17	**53**	**82**	**149**	11	**7**	13	3	11	22	**603**	3	228	475	1	499	**4**
18	**201**	**352**	n/av	21	n/av	n/av	**0**	10	**8**	539	3	259	n/av	n/av	n/av	n/av
19	**64**	**85**	122	13	2	16	**2**	10	24	437	2	214	462	0	437	0
20	64	**164**	**240**	27	5	15	2	8	**15**	477	**8**	256	497	0	527	1
21	**150**	**400**	**420**	**36**	**18**	**0**	2	**4**	**12**	**720**	3	320	**634**	0	650	**7**
22	**52**	**71**	**167**	16	1	12	3	11	**12**	534	2	285	558	0	600	0
23	**153**	**292**	**484**	19	6	**7**	3	10	**12**	**593**	2	274	**609**	1	**700**	3
24	**188**	**173**	**378**	**36**	**19**	9	3	7	**14**	498	3	214	456	1	484	2
25	**349**	n/ad	n/ad	**36**	n/av	n/av	n/av	6	**9**	360	**14**	256	n/av	n/av	n/av	n/av
26	**63**	**54**	**155**	13	2	15	**2**	11	**13**	403	4	233	497	0	493	0
27	**111**	**122**	**240**	20	4	**7**	**1**	9	**18**	380	1	241	529	0	555	1
28	55	**131**	**188**	14	3	12	**1**	7	20	542	3	203	542	0	568	0
29	**78**	**135**	**227**	26	5	7	3	9	**15**	437	1	225	511	1	555	1
30	91	107	**232**	16	**14**	6	**1**	5	**10**	596	1	**399**	a	a	a	a
31	**231**	**391**	**a 393**	**38**	a	**3**	**0**	**3**	**7**	532	5	272	594	**3**	**832**	**10**
32	**94**	**207**	**195**	21	6	14	3	9	24	**550**	0	266	584	0	**635**	0
33	34	**85**	**178**	16	0	12	3	8	29	505	1	244	494	0	505	0
34	54	67	155	16	6	14	**1**	10	19	371	4	231	492	0	507	1
35	32	**65**	127	14	0	14	3	11	26	421	2	219	474	0	487	0
36	**138**	**189**	**494**	17	3	**3**	3	10	**8**	373	0	247	484	0	479	0
37	**74**	**131**	**312**	**31**	**18**	10	**1**	10	**16**	374	1	254	518	1	556	**4**
38	**167**	**197**	**a 360**	28	**14**	14	**0**	**4**	**6**	529	**6**	224	526	0	541	3
n/imp	38/25	36/32	34/29	38/10	32/11	36/10	36/21	38/4	37/25	36/6	36/8	37/2	31/2	31/1	31/6	31/4
more imp	84.2	94.1	100	47.4	78.6	58.8	61.1	21.1	89.5	27.8	33.3	10.5	15.4	7.7	30.8	23.1
less imp	47.4	84.2	73.7	5.3	0	0	55.6	0	44.4	5.6	11.1	0	0	0	11.1	5.6

Notes: n/av = not available (organisational), n/ad = not administered (based on performance in other tests), a = abandoned (stopped after instruction or during test), imp = impaired, bold = score below cut-off thus considered as impaired¸ more/less impaired indicates the percentage of participants with impaired scores for the two subgroups of more/less impaired patients based on a median split of nonverbal severity

Supplementary Table 3. Participants' scores in language tests.

	Digit Span		Repetition immediate		Repetition delayed		Naming		Minimal Pairs		CCT	Synonym	Word-picture matching		Compre-hension	Cookie Theft		
Subj	forward	backward	W	NW	W	NW	Cambridge	Boston	W	NW	pictures	judgment	Spoken	Written	Sentence	Token	MLU	WPM
1	**3**	**0**	**59**	18	55	3	**48**	**43**	67	**58**	63	92	63	63	**18**	38	**6.86**	32.6
2	7	6	80	27	79	27	60	53	72	**66**	**51**	**90**	64	64	**27**	60	11.8	212
3	5	4	80	26	77	24	61	**40**	68	67	**47**	**80**	63	**60**	**25**	38	**8.17**	50.7
4	8	7	**62**	22	70	25	**56**	**47**	71	72	**48**	**86**	64	64	28	**30**	**7.4**	18
5	**2**	**0**	**30**	0	0	0	**0**	**0**	**38**	**16**	**17**	**47**	**37**	**20**	**4**	*32*	**8.2**	87.3
6	**4**	4	80	30	80	27	**57**	**33**	70	71	58	**87**	64	64	28	56	14.8	56
7	**3**	**2**	**40**	8	49	5	**30**	**23**	71	71	**38**	**55**	**62**	**60**	**4**	*33*	**3.18**	7.42
8	**3**	**2**	**75**	16	33	3	**56**	**30**	69	**65**	59	**75**	64	**62**	**20**	47	10	49.5
9	6	**3**	**46**	7	44	4	**34**	**30**	67	69	55	**79**	**62**	63	**22**	**25**	**4**	25.4
10	**4**	4	**72**	15	71	14	**57**	**26**	67	**54**	61	93	64	64	**26**	315	19.6	106
11	5	**3**	**35**	7	28	2	**25**	**6**	67	69	**48**	**63**	64	**62**	**11**	**11**	**1.4**	5.16
12	7	**3**	**77**	25	77	21	**54**	**37**	69	69	**54**	**76**	64	64	**25**	74	13	92.5
13	**2**	**2**	**41**	3	34	1	**35**	**14**	**62**	**64**	59	**79**	63	64	**18**	**31**	**6.83**	29.5
14	**3**	**2**	**76**	13	76	14	**46**	**34**	69	71	60	**86**	63	64	**25**	94	11.9	56.4
15	**0**	**0**	**1**	0	0	0	**0**	**0**	**56**	**59**	**43**	**72**	**50**	**60**	**15**	**0**	**0**	0
16	**3**	**2**	**69**	8	51	4	**49**	**25**	**63**	**58**	**51**	**72**	**61**	63	**24**	122	16.6	56.3
17	**3**	**0**	**31**	0	19	0	**22**	**9**	**58**	**57**	59	**86**	64	64	**23**	38	**3.15**	27.5
18	7	**2**	**52**	10	53	5	**42**	**23**	**59**	**62**	**52**	**82**	**62**	63	29	**18**	**8**	17.1
19	**4**	**2**	**74**	18	71	12	**54**	**36**	70	69	58	**84**	64	64	**27**	38	**6.83**	23.8
20	6	**0**	**68**	17	73	13	**39**	**23**	68	**58**	57	**80**	64	64	28	**25**	**4.71**	19.7
21	**2**	**0**	**0**	0	0	0	**3**	**3**	**61**	**53**	**50**	**71**	64	**61**	**18**	46	**5.22**	15.3
22	5	4	80	26	80	24	60	53	71	71	**54**	**83**	63	63	29	**22**	**10.3**	69.5
23	**3**	**0**	**71**	16	31	6	**9**	**2**	**62**	70	**42**	**45**	**40**	**59**	**15**	58	**5.17**	32.8
24	**2**	**0**	**4**	0	0	0	**1**	**0**	69	**66**	**38**	**47**	**50**	**36**	**16**	**1**	**0.17**	1
25	**2**	**0**	**50**	5	26	1	**16**	**10**	**31**	**34**	**34**	**44**	**55**	**39**	**14**	**12**	**4.75**	7.5
26	**4**	**2**	**70**	21	58	20	**56**	**44**	71	71	58	**84**	64	64	30	48	14.5	44.3
27	5	**0**	**76**	18	75	17	**54**	**46**	66	**59**	**53**	**85**	63	63	**26**	**23**	**4.67**	37.3
28	**2**	**2**	**11**	0	6	1	**4**	**3**	71	67	57	**67**	64	63	**19**	55	9.29	110
29	**0**	**0**	**0**	0	0	0	**0**	**0**	**62**	**61**	**40**	**50**	**38**	**42**	**11**	**0**	**0**	0
30	5	**3**	**61**	9	57	3	**35**	**33**	**55**	**57**	**48**	91	63	63	**24**	122	17	97.6
31	**2**	**2**	**52**	3	44	4	**38**	**31**	67	**66**	**42**	**67**	**59**	63	**20**	**19**	**3.29**	19.7
32	**2**	**2**	**17**	1	4	1	**2**	**1**	69	69	57	**86**	**62**	**62**	**20**	203	19	94.4
33	**4**	**3**	**73**	24	79	14	**56**	**47**	67	**62**	59	92	**61**	**62**	30	116	16.8	47.7
34	5	**3**	**57**	14	54	9	**50**	**33**	66	**63**	56	**84**	64	64	**27**	69	11.6	55.2
35	5	**3**	**68**	15	72	11	**50**	**46**	70	**63**	59	92	64	64	32	94	13.6	49.5
36	5	**3**	79	29	76	13	**58**	51	68	69	61	**77**	64	64	**20**	74	**6.69**	74
37	**0**	**0**	**0**	0	0	0	**0**	**0**	**51**	**54**	**46**	**67**	**34**	**58**	**14**	**0**	**0**	0
38	**2**	**2**	**40**	2	23	2	**31**	**14**	**40**	**33**	**26**	**67**	**54**	**59**	**14**	73	11.7	46.6
Total impaired	24	32	33	-	-	-	35	35	13	23	21	33	15	15	30	14	22	-

Notes: bold = impaired; - in the last row indicates that no norms were available

	1	2	3	4	5	6	7	8	9	10	11	12	13	14	15	16	17	18	19	20	21	22	23	24	25	26	27	28	29	30	31	32	33	34
1 Trail making numbers		0.80	0.76	0.42	0.67	0.59	0.42	0.57	0.64	0.56	0.47	0.35	0.30	0.44	0.44	0.59	0.20	0.28	0.22	0.33	0.33	0.31	0.37	0.33	0.51	0.59	0.72	0.58	0.42	0.72	0.66	0.21	0.23	0.06
2 Trail making letters	0.80		0.75	0.52	0.52	0.61	0.27	0.07	0.49	0.54	0.53	0.30	0.62	0.45	0.71	0.76	0.16	0.27	0.32	0.39	0.38	0.40	0.40	0.41	0.24	0.10	0.40	0.46	0.18	0.12	0.37	0.17	0.34	0.36
3 Trail making switching	0.76	0.75		0.45	0.59	0.70	0.10	0.18	0.30	0.63	0.20	0.18	0.20	0.20	0.30	0.43	0.19	0.27	0.26	0.14	0.38	0.29	0.39	0.36	0.26	-0.06	0.50	0.63	0.38	0.30	0.59	0.32	0.52	0.40
4 Brixton correct	0.42	0.52	0.45		0.62	0.53	0.29	0.46	0.52	0.41	0.12	0.01	0.26	0.38	0.45	0.60	0.35	0.35	0.47	0.49	0.52	0.48	0.46	0.42	0.42	0.41	0.52	0.52	0.40	0.49	0.54	0.36	0.50	0.36
5 Divided accuracy	0.67	0.52	0.59	0.62		0.42	0.23	0.35	0.47	0.42	0.24	0.20	0.05	0.27	0.18	0.48	0.23	0.23	0.40	0.41	0.49	0.43	0.39	0.42	0.35	0.27	0.53	0.45	0.21	0.48	0.57	0.24	0.37	0.28
6 TOL planning	0.59	0.61	0.70	0.53	0.42		0.37	0.43	0.59	0.52	0.42	0.38	0.35	0.38	0.50	0.52	0.08	0.18	0.23	0.17	0.25	0.26	0.28	0.14	0.18	0.17	0.42	0.44	0.24	0.36	0.57	0.31	0.45	0.17
7 Kramer categories	0.42	0.27	0.10	0.29	0.23	0.37		0.51	0.60	0.57	0.25	0.25	-0.03	0.23	0.14	0.30	-0.04	0.04	0.22	0.26	0.16	0.14	0.19	0.15	0.39	0.23	0.53	0.26	0.13	0.11	0.29	0.48	0.32	0.17
8 GoNoGo accuracy	0.57	0.07	0.18	0.46	0.35	0.43	0.51		0.62	0.53	0.22	0.23	-0.15	0.22	0.07	0.23	-0.03	0.06	0.03	0.22	0.17	0.20	0.17	0.16	0.59	0.52	0.54	0.35	0.21	0.47	0.30	0.21	0.11	0.10
9 Raven B	0.64	0.49	0.30	0.52	0.47	0.59	0.60	0.62		0.59	0.50	0.35	0.29	0.29	0.43	0.58	0.10	0.13	0.29	0.37	0.30	0.29	0.34	0.25	0.40	0.45	0.70	0.49	0.20	0.45	0.50	0.20	0.18	0.07
10 Design Fluency total	0.56	0.54	0.63	0.41	0.42	0.52	0.57	0.53	0.59		0.29	0.36	0.15	0.31	0.27	0.42	0.10	0.17	0.18	0.28	0.28	0.27	0.26	0.30	0.42	0.27	0.65	0.63	0.28	0.35	0.50	0.35	0.32	0.28
11 GoNoGo RT	0.47	0.53	0.20	0.12	0.24	0.42	0.25	0.22	0.50	0.29		0.32	0.40	0.20	0.37	0.45	0.13	0.16	0.30	0.36	0.34	0.27	0.41	0.41	0.20	0.26	0.35	0.20	0.13	0.24	0.31	-0.02	0.00	-0.07
12 Alertness RT	0.35	0.30	0.18	0.01	0.20	0.38	0.25	0.23	0.35	0.36	0.32		0.46	0.12	0.47	0.35	0.02	-0.04	0.06	0.07	-0.03	0.08	0.04	0.02	-0.11	-0.14	0.21	0.21	-0.09	-0.05	0.39	0.03	0.13	0.13
13 Distract RT w/o distractor	0.30	0.62	0.20	0.26	0.05	0.35	-0.03	-0.15	0.29	0.15	0.40	0.46		0.11	0.86	0.40	0.00	-0.02	0.13	0.15	0.17	0.13	0.22	0.16	0.00	-0.04	0.11	0.17	0.02	-0.16	0.24	-0.04	0.00	0.02
14 Distract corr w/o distractor	0.44	0.45	0.20	0.38	0.27	0.38	0.23	0.22	0.29	0.31	0.20	0.12	0.11		0.50	0.71	0.34	0.19	0.18	0.25	0.22	0.17	0.24	0.23	0.12	-0.07	0.39	0.42	0.40	0.32	0.26	0.29	0.40	0.31
15 Distract RT w distractor	0.44	0.71	0.30	0.45	0.18	0.50	0.14	0.07	0.43	0.27	0.37	0.47	0.86	0.50		0.62	0.09	0.03	0.15	0.21	0.22	0.20	0.24	0.18	0.01	-0.06	0.27	0.30	0.13	-0.05	0.32	0.09	0.18	0.15
16 Distract corr w distractor	0.59	0.76	0.43	0.60	0.48	0.52	0.30	0.23	0.58	0.42	0.45	0.35	0.40	0.71	0.62		0.38	0.36	0.36	0.49	0.41	0.42	0.37	0.35	0.39	0.31	0.44	0.39	0.26	0.13	0.36	0.27	0.44	0.36
17 Digit Span forward	0.20	0.16	0.19	0.35	0.23	0.08	-0.04	-0.03	0.10	0.10	0.13	0.02	0.00	0.34	0.09	0.38		0.67	0.68	0.69	0.76	0.68	0.71	0.70	0.42	0.36	0.36	0.52	0.60	0.42	0.63	0.10	0.31	0.34
18 Digit Span backward	0.28	0.27	0.27	0.35	0.23	0.18	0.04	0.06	0.13	0.17	0.16	-0.04	-0.02	0.19	0.03	0.36	0.67		0.53	0.60	0.62	0.69	0.66	0.64	0.46	0.42	0.29	0.52	0.52	0.44	0.50	0.32	0.50	0.51
19 Repetition immediate W	0.22	0.32	0.26	0.47	0.40	0.23	0.22	0.03	0.29	0.18	0.30	0.06	0.13	0.18	0.15	0.36	0.68	0.53		0.86	0.92	0.74	0.90	0.83	0.34	0.28	0.41	0.50	0.52	0.42	0.63	0.27	0.52	0.38
20 Repetition immediate NW	0.33	0.39	0.14	0.49	0.41	0.17	0.26	0.22	0.37	0.28	0.36	0.07	0.15	0.25	0.21	0.49	0.69	0.60	0.86		0.88	0.89	0.85	0.85	0.50	0.44	0.47	0.52	0.46	0.42	0.63	0.14	0.40	0.37
21 Repetition delayed W	0.33	0.38	0.38	0.52	0.49	0.25	0.16	0.17	0.30	0.28	0.34	-0.03	0.17	0.22	0.22	0.41	0.76	0.62	0.92	0.88		0.83	0.93	0.91	0.49	0.41	0.51	0.64	0.61	0.53	0.72	0.25	0.46	0.33
22 Repetition delayed NW	0.31	0.40	0.29	0.48	0.43	0.26	0.14	0.20	0.29	0.27	0.27	0.08	0.13	0.17	0.20	0.42	0.68	0.69	0.74	0.89	0.83		0.77	0.78	0.50	0.42	0.35	0.51	0.43	0.38	0.67	0.13	0.39	0.41
23 Naming Cambridge	0.37	0.40	0.39	0.46	0.39	0.28	0.19	0.17	0.34	0.26	0.41	0.04	0.22	0.24	0.24	0.37	0.71	0.66	0.90	0.85	0.93	0.77		0.93	0.48	0.37	0.54	0.68	0.67	0.56	0.72	0.24	0.49	0.32
24 Naming Boston	0.33	0.41	0.36	0.42	0.42	0.14	0.15	0.16	0.25	0.30	0.41	0.02	0.16	0.23	0.18	0.35	0.70	0.64	0.83	0.85	0.91	0.78	0.93		0.49	0.39	0.49	0.68	0.61	0.50	0.70	0.14	0.42	0.36
25 Minimal Pairs W	0.51	0.24	0.26	0.42	0.35	0.18	0.39	0.59	0.40	0.42	0.20	-0.11	0.00	0.12	0.01	0.39	0.42	0.46	0.34	0.50	0.49	0.50	0.48	0.49		0.88	0.69	0.48	0.55	0.59	0.50	0.16	0.18	0.19
26 Minimal Pairs NW	0.59	0.10	-0.06	0.41	0.27	0.17	0.23	0.52	0.45	0.27	0.26	-0.14	-0.04	-0.07	-0.06	0.31	0.36	0.42	0.28	0.44	0.41	0.42	0.37	0.39	0.88		0.64	0.36	0.46	0.65	0.43	0.00	0.04	0.01
27 Camel & Cactus	0.72	0.40	0.50	0.52	0.53	0.42	0.53	0.54	0.70	0.65	0.35	0.21	0.11	0.39	0.27	0.44	0.36	0.29	0.41	0.47	0.51	0.35	0.54	0.49	0.69	0.64		0.77	0.67	0.76	0.69	0.33	0.36	0.19
28 Synonym judgment	0.58	0.46	0.63	0.52	0.45	0.44	0.26	0.35	0.49	0.63	0.20	0.21	0.17	0.42	0.30	0.39	0.52	0.52	0.50	0.52	0.64	0.51	0.68	0.68	0.48	0.36	0.77		0.69	0.73	0.80	0.43	0.57	0.38
29 Word-picture matching spoken	0.42	0.18	0.38	0.40	0.21	0.24	0.13	0.21	0.20	0.28	0.13	-0.09	0.02	0.40	0.13	0.26	0.60	0.52	0.52	0.46	0.61	0.43	0.67	0.61	0.55	0.46	0.67	0.69		0.69	0.62	0.29	0.44	0.28
30 Word-picture matching written	0.72	0.12	0.30	0.49	0.48	0.36	0.11	0.47	0.45	0.35	0.24	-0.05	-0.16	0.32	-0.05	0.13	0.42	0.44	0.42	0.42	0.53	0.38	0.56	0.50	0.59	0.65	0.76	0.73	0.69		0.61	0.26	0.31	0.15
31 Sentence comprehension	0.66	0.37	0.59	0.54	0.57	0.57	0.29	0.30	0.50	0.50	0.31	0.39	0.24	0.26	0.32	0.36	0.63	0.50	0.63	0.63	0.72	0.67	0.72	0.70	0.50	0.43	0.69	0.80	0.62	0.61		0.29	0.54	0.29
32 Cookie Theft Token	0.21	0.17	0.32	0.36	0.24	0.31	0.48	0.21	0.20	0.35	-0.02	0.03	-0.04	0.29	0.09	0.27	0.10	0.32	0.27	0.14	0.25	0.13	0.24	0.14	0.16	0.00	0.33	0.43	0.29	0.26	0.29		0.80	0.52
33 Cookie Theft MLU	0.23	0.34	0.52	0.50	0.37	0.45	0.32	0.11	0.18	0.32	0.00	0.13	0.00	0.40	0.18	0.44	0.31	0.50	0.52	0.40	0.46	0.39	0.49	0.42	0.18	0.04	0.36	0.57	0.44	0.31	0.54	0.80		0.65
34 Cookie Theft WPM	0.06	0.36	0.40	0.36	0.28	0.17	0.17	0.10	0.07	0.28	-0.07	0.13	0.02	0.31	0.15	0.36	0.34	0.51	0.38	0.37	0.33	0.41	0.32	0.36	0.19	0.01	0.19	0.38	0.28	0.15	0.29	0.52	0.65	

Supplementary Fig. 1. Pairwise Pearson correlations between all raw scores. Some scores have been transformed so that a higher score always corresponds to better performance (e.g. by taking the inverse of reaction time measures).

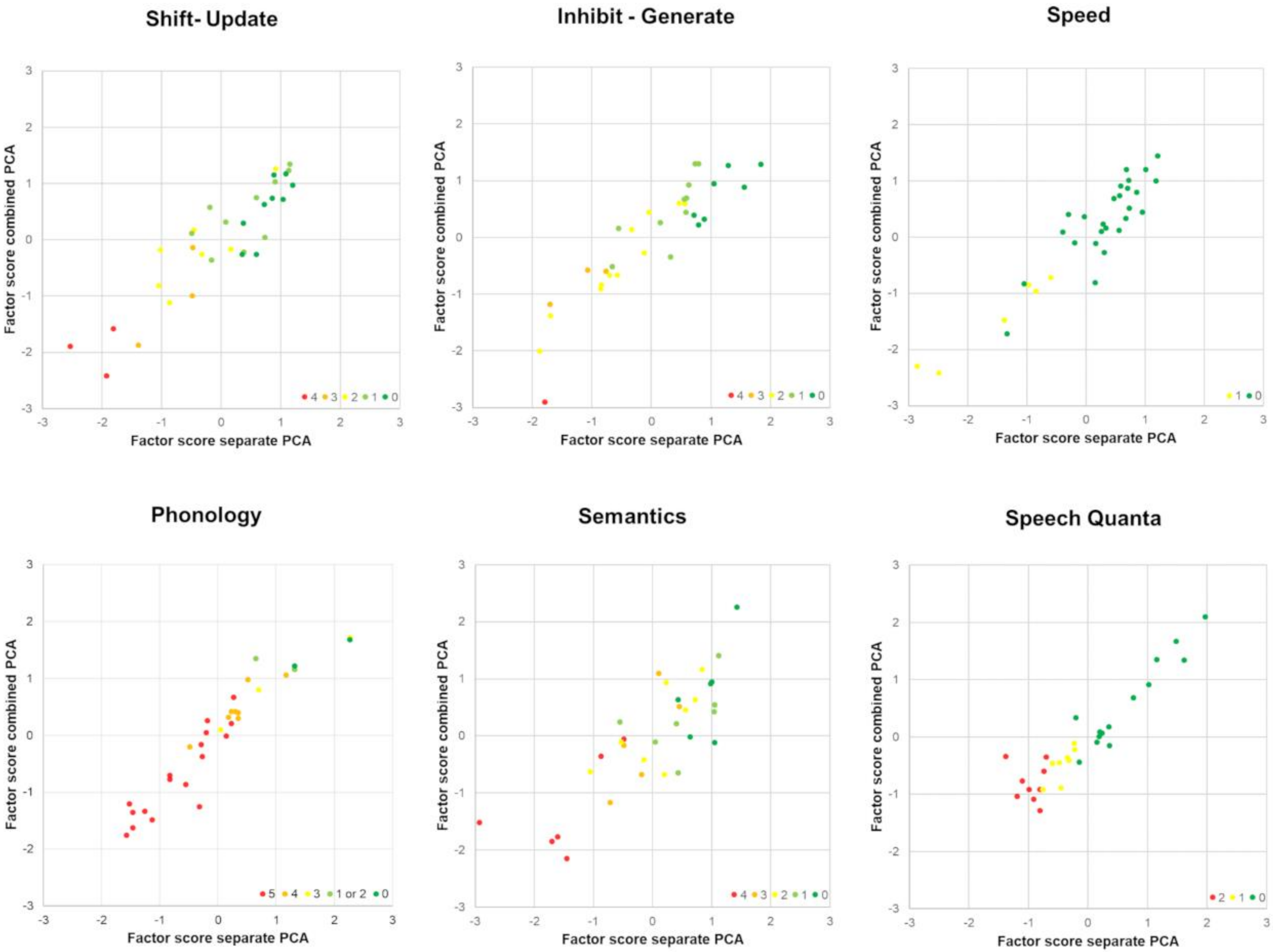
Shift- Update
Inhibit - Generate
Speed
Phonology
Semantics
Speech Quanta
Factor score combined PCA
Factor score separate PCA
4 3 2 1 0
2 1 0
5 4 3 1 or 2 0

Supplementary Fig. 2. Relationship between and individual’s separate and combined component scores and impaired test performance. The colour (legend in the bottom right corner of each graph) indicates the level of impairment (i.e. the individual’s number of impaired test scores) in the tests contributing most to each component of the separate analyses (Shift-Update: Trails numbers, Brixton, Divided omissions, Tower of London; Inhibit-Generate: Kramer, Raven B, GoNoGo errors, Design Fluency; Speed: Alertness and GoNoGo median reaction time; Phonology: Boston and Cambridge Naming, Digit Spans, Repetition; Semantics: Synonym judgment, Camel & Cactus, spoken and written Word-Picture matching; Speech Quanta: Token and mean length of utterance of Cookie Theft; see also Fig. 4 in main text). A high correlation between the two component scores (see also Table 1 in main text) and a relatively good fit between component score and impairment can be observed (note that raw scores (sometimes combined) were included in the PCA while impairment in a test was based on norm data, which was corrected for age and education for some tests). Impairment in the Minimal Pairs and in spoken sentence comprehension is not considered as both tests loaded nearly identically on both language components in the separate analyses.

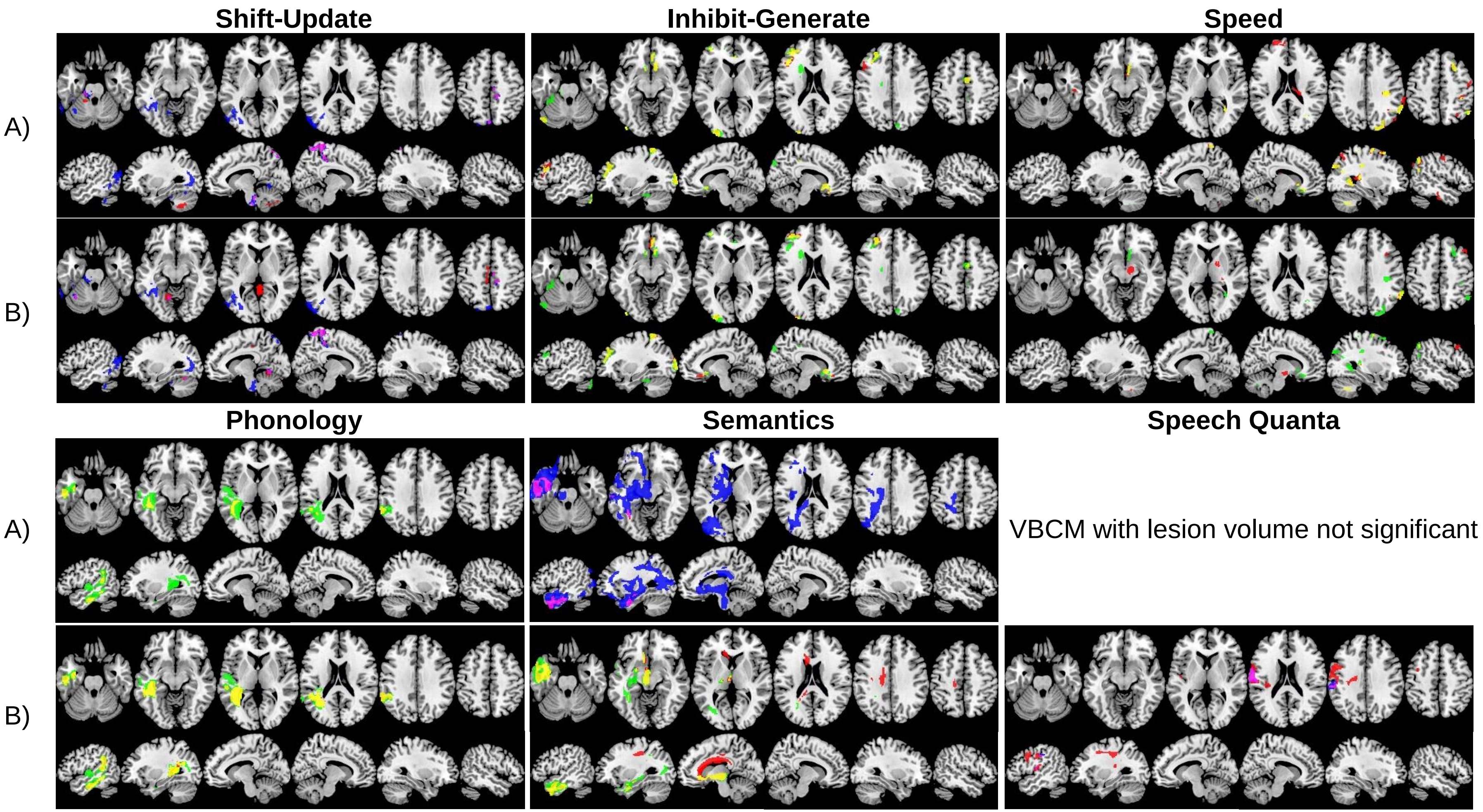

Shift-Update
Inhibit-Generate
Speed
A)
B)
Phonology
Semantics
Speech Quanta
A)
VBCM with lesion volume not significant
B)

Supplementary Fig. 3. Comparison of VBCM clusters with and without covariates. (A) shows the overlap of the VBCM clusters without covariates with the VBCM clusters including lesion as a covariate. (B) shows the comparison with the VBCM clusters including age, education, time post stroke as covariates; The significant VBCM clusters without covariates, presented in the main text, are shown in blue (voxel-level threshold 0.01) or green (voxel-level threshold 0.001), the clusters they are compared to (same voxel-level threshold) are shown in red, their overlap in magenta or yellow, respectively. The cluster-level threshold was set at $p < 0.05$, FWEc. MNI coordinates from left to right are z = -25, -10, 5, 20, 35, 50 for the axial slices and x = -50, -30, -10, 10, 30, 50 for the sagittal slices. Figures are in neurological convention (left is left) and thresholded at the respective minimum t-value.

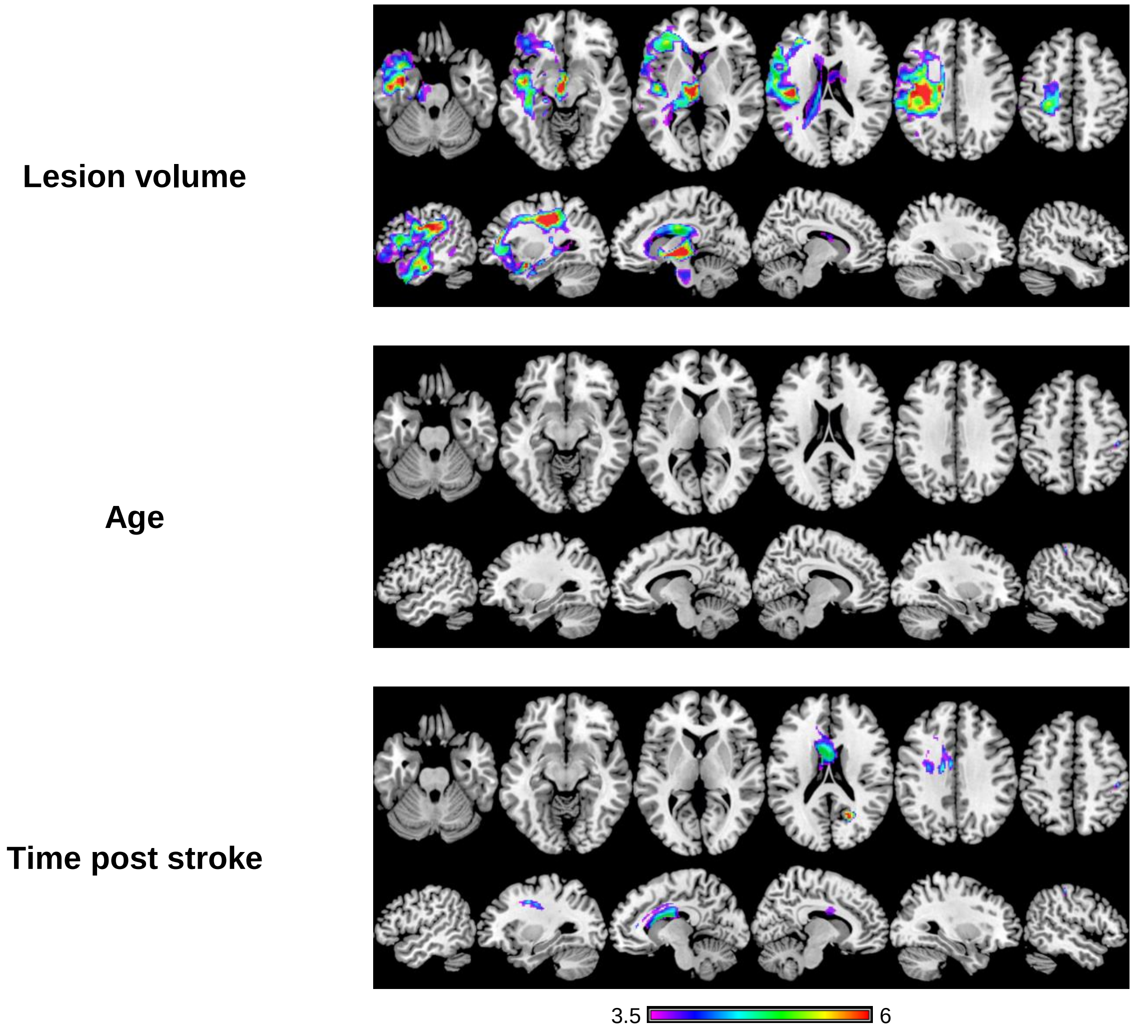

Supplementary Fig. 4. Cluster associated with lesion volume, age, and time post stroke when only the patient characteristics were included in the VBCM analysis. There was no cluster associated with education at the applied threshold (voxel-level $p < 0.001$ and FWEc at cluster-level $p \leq 0.05$). The same slices as in Supplementary Fig. 3 are shown. Figures are in neurological convention (left is left).

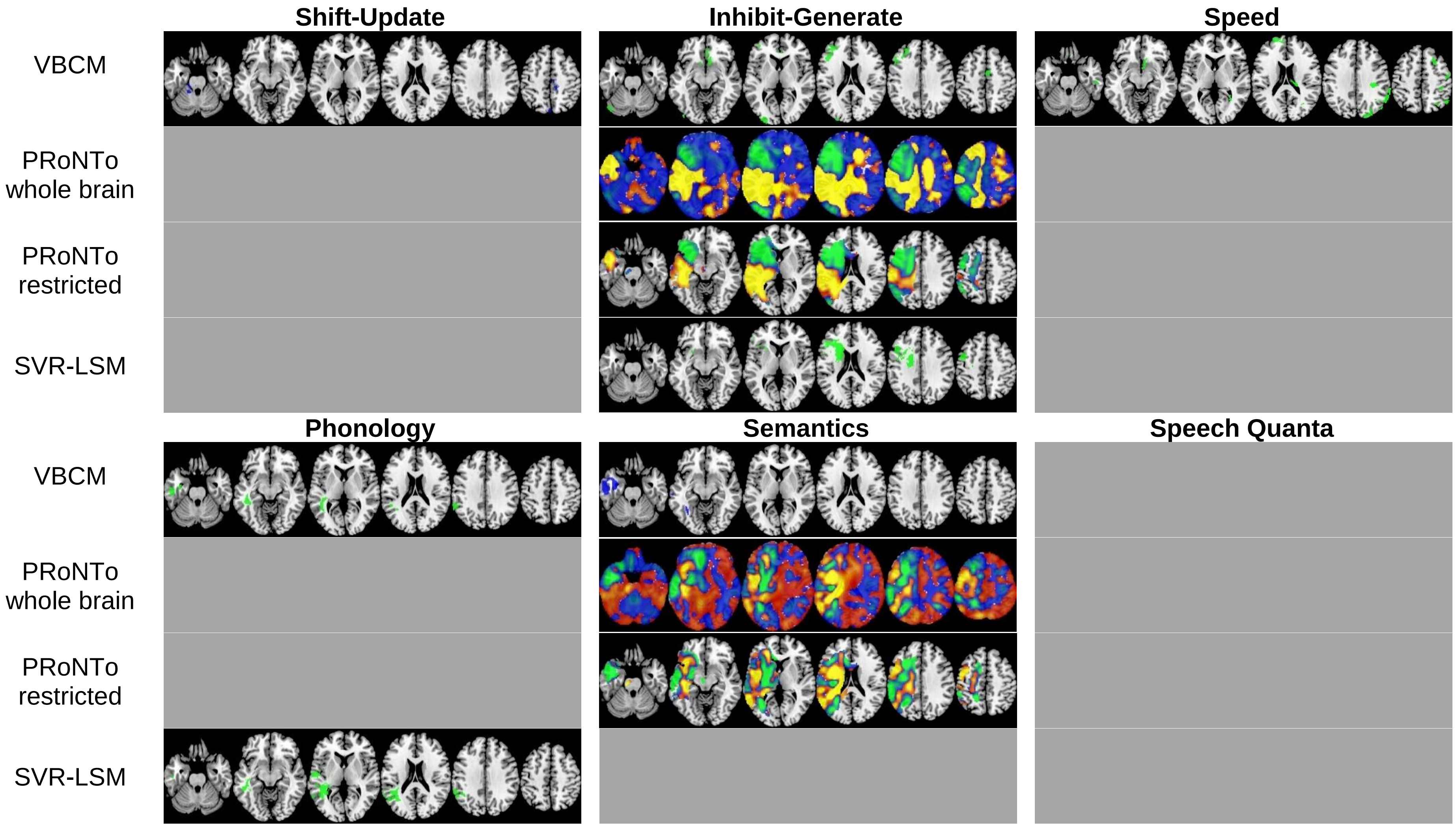
Shift-Update
Inhibit-Generate
Speed
VBCM
PRoNTo whole brain
PRoNTo restricted
SVR-LSM
Phonology
Semantics
Speech Quanta

Supplementary Fig. 5. Comparison of the univariate and multivariate approaches when lesion volume is included as covariate. The VBCM clusters are shown in blue (voxel-level threshold 0.01) and green (voxel-level threshold 0.001), a family-wise error correction of $p \leq 0.05$ was applied to all clusters, and images are thresholded at the respective minimum t-value. The PRoNTo results depict the weights for the winning model if significant (see text), either including the whole brain space or restricting it to lesion territory ($N > 3$). They are thresholded from -0.005 to -0.0001 (green-blue) and 0.0001 to 0.005 (red-yellow). The negative weights are considered as more meaningful in this approach. The SVR-LSM images show voxels with significant beta weights after permutation testing (N=10,000, voxel-wise $p < 0.005$ and cluster-wise $p < 0.05$). MNI coordinates of slices, from left to right, are z = -25, -10, 5, 20, 35, 50 and they are in neurological convention (left is left). A grey surface indicates that no significant results were found for the respective component and methodological approach.

Effects of including patient characteristics as covariates

When lesion volume was included in the VBCM analysis, the extent of the clusters was generally reduced, as shown in Supplementary Fig. 3. Also, the speech quanta cluster was not significant anymore and the semantics cluster only appeared at a less strict threshold. Supplementary Fig. 4 shows that brain regions on the edge of the overall lesion extent were associated with lesion volume in the VBCM that included the patient characteristics only. Peak regions of this lesion volume cluster overlap with the semantics and speech quanta components, which explains why they emerged only at a less strict threshold or not anymore when lesion volume was taken into account. Similarly, when lesion volume was included in the multivariate approaches, as shown in Supplementary Fig. 5, only the models for inhibit-generate and for phonology remained significant in the SVR-LSM. Interestingly, the SVR-LSM result for inhibit-generate is more similar to the (uncorrected) VBCM cluster if lesion volume is added as a covariate. The PRoNTo approaches yielded significant results for inhibit-generate (KRR model whole brain: cross-validation $r = 0.338$, $MSE = 0.857$, $p = 0.028$; KRR model restricted lesion territory: $r = 0.384$, $MSE = 0.818$, $p = 0.028$) and semantics (KRR model whole brain: $r = 0.603$, $MSE = 0.402$, $p < 0.002$; KRR model restricted lesion territory: $r = 0.597$, $MSE = 0.419$, $p < 0.001$) only. Including lesion volume as a covariate has thus not the same effect in the different brain-behaviour mapping approaches, but generally leads to a reduction of significant models. It remains possible that including lesion volume in brain-behaviour mapping might lead to Type II error; hence it is unclear which strategy might be optimal.

The other patient characteristics (age, education, time post stroke) had a weaker effect on the brain-behaviour mapping. All nonverbal and verbal clusters still emerged at the same thresholds as when analysed by means of the VBCM without the covariates. However, their extent was somewhat smaller but could also include additional areas.

References

Butler RA, Lambon Ralph MA, Woollams AM. Capturing multidimensionality in stroke aphasia: mapping principal behavioural components to neural structures. Brain 2014; 137: 3248-66.

Dronkers NF, Larsen JL. Neuroanatomy of the classical syndromes of aphasia. In: Boller F, Grafman J, editors. Handbook of Neuropsychology. 2nd ed. New York: Elsevier Science; 2001. p. 19-30.

Kasselimis DS, Simos PG, Peppas C, Evdokimidis I, Potagas C. The unbridged gap between clinical diagnosis and contemporary research on aphasia: A short discussion on the validity and clinical utility of taxonomic categories. Brain and Language 2017; 164: 63-7.
